# Editorial: Phospholipids and sphingolipids in nutrition, metabolism, and health

**DOI:** 10.3389/fnut.2023.1153138

**Published:** 2023-02-07

**Authors:** Ronan Lordan, Christopher N. Blesso

**Affiliations:** ^1^Perelman School of Medicine, Institute for Translational Medicine and Therapeutics, University of Pennsylvania, Philadelphia, PA, United States; ^2^Department of Medicine, Perelman School of Medicine, University of Pennsylvania, Philadelphia, PA, United States; ^3^Department of Systems Pharmacology and Translational Therapeutics, Perelman School of Medicine, University of Pennsylvania, Philadelphia, PA, United States; ^4^Department of Nutritional Sciences, University of Connecticut, Storrs, CT, United States

**Keywords:** phospholipids, sphingolipids, glycolipids, gangliosides, inflammation, lipid metabolism

Phospholipids and sphingolipids are ubiquitous in nature. Often referred to as polar lipids, these lipids are essential components in cell membranes and consist of a hydrophilic head group and a hydrophobic tail. Glycerophospholipids share a common structure composed of two hydrophobic fatty acid molecules esterified to the *sn*-1 and *sn*-2 positions of the glycerol moiety and a hydrophilic phosphate group bound to the *sn*-3 position. A substituted head group is attached to the phosphate and or with phosphodiester linkages to organic molecules including choline (phosphatidylcholine), ethanolamine (phosphatidylethanolamine), inositol (phosphatidylinositol), or serine (phosphatidylserine). The most abundant phospholipids in animal tissues tend to be phosphatidylcholine (PC) and phosphatidylethanolamine (PE). On the other hand, in sphingolipids the glycerol component is replaced by a long-chain amino alcohol known as sphingosine, which is amide-linked to a fatty acid and phosphate group. The most common example of a sphingolipid is sphingomyelin (SM), which contains a sphingosine and a choline molecule (1). These structural elements are what confer the amphipathic properties that give these lipids their critical functions in providing structure and fluidity to cell membranes. However, these molecules also play an important role in cell signaling as secondary messengers and as precursors for bioactive lipids such as prostaglandins and platelet-activating factor (PAF) with many health consequences (1, 2). These lipids also provide energy and may confer health effects upon ingestion of food (3).

The aim of this Research Topic was to collate state-of-the-art original research articles and reviews regarding the role of phospholipids and sphingolipids in the fields of nutrition, metabolism, and health. Over the past few decades, there has been considerable research interest in the role of dietary phospholipids and sphingolipids with regards to diseases characterized by inflammation. On this trail of thought, Miklavcic et al. focused on the role of dietary gangliosides in attenuating pro-inflammatory signaling in inflammatory bowel disease (IBD) models. They assessed the effect of milk powder-derived gangliosides administered to Caco-2 intestinal epithelial cells *in vitro* on tight junction integrity and inflammatory signaling in response to pro-inflammatory stimuli. Gangliosides are a class of polar lipid present in abundance in nervous tissue but also present in other body tissues and can be obtained in the diet from milk and other animal sources (1, 4). They found that gangliosides improved intestinal integrity by modulating secretory phospholipase A_2_ (sPLA_2_) trafficking and the synthesis of pro-inflammatory mediators such as human beta-defensin 2 (HBD-2) and IL-23 *via* inhibiting nuclear factor-κB (NF-κB) assembly. These findings point to the need for further research to determine whether gangliosides may provide prophylactic or therapeutic benefits in conditions characterized by poor intestinal tight junction integrity as observed in IBD.

Other notable bioactive lipids present in milk in low abundance are conjugated linoleic acid (CLA) and docosahexaenoic acid (DHA). Murru et al. investigated whether maternal intake of a supplemented diet of CLA and DHA in phospholipid form (CLA-DHA-PL) would affect maternal fetal brain and liver fatty acid and the *N*-acylethanolamine profiles, which are important for central nervous system (CNS) development. They fed rat dams a specialized diet containing 0.5% w/w CLA and 0.2% DHA bound to phospholipids during the first $\frac{2}{3}$ of pregnancy. The authors determined that CLA and DHA in the polar lipid form readily crossed the placenta and were incorporated in the fetal liver and brains. The diet also affected the biosynthesis of *N*-acylethanolamines derived from arachidonic acid and DHA such as *N*-arachidonoylethanolamine and *N*-docosahexaenoylethanolamine (DHEA), respectively. Indeed, the authors suggest that CLA-DHA-PL supplementation in mothers may promote CNS development as increased DHEA may promote the synthesis of neurites and synapses, which may lower the risk for the development of neurological disorders in the offspring. Although in humans findings regarding supplementation of DHA incorporated into polar lipids alone show mixed findings regarding neurodevelopment (5). However, as Murru et al. demonstrate, there may be additional benefits of combining CLA and DHA in the polar lipid form. Therefore, CLA-DHA-PL should be further investigated in humans.

Another supplementation study in this Research Topic conducted by Sugimoto et al. investigated the effects of an oil (SCO-PL) obtained from the Japanese giant scallop (*Patinopecten yessoensis*) that is high in dietary phospholipids and eicosapentaenoic acid (EPA) on the serum and liver cholesterol contents in mice. Mice were supplemented with either SCO-PL (high in polar lipids) or a triglyceride oil (SCO-TG) with similar fatty acid compositions along with soybean oils high in either polar lipids (SOY-PL) or triglycerides (SOY-TG). At 5 weeks old, mice were split into groups and fed a high fat diet containing 3% w/w of one of the experimental oils for 28 days. The authors found that the SCO-PL diet significantly decreased serum and liver cholesterol compared to the SOY-TG diet, but similar effects were not observed with the intake of SOY-PL or SCO-TG. The authors further determined that the SCO-PL benefits were in part because of increased expression of the rate-limiting enzyme for bile synthesis in the liver (cholesterol 7a-hydoxylase, *Cyp7a1*), and reduction of expression of the farnesoid X receptor (*Fxr*) and fibroblast growth factor 15 (*Fgf15*) in the ileum, which inhibit the expression of liver *Cyp7a1*. Both the SCO-PL and SOY-PL exhibited increased fecal neutral sterol excretion vs. the TG diets. Further structure-function studies are required to determine what polar lipids in these mixtures are responsible for these biological effects, which may be important for the development of future therapeutics or nutraceuticals.

Indeed, polar lipids from multiple sources such as milk appear to exhibit cholesterol-lowering effects and benefits against cardiovascular risk factors (3). Calzada et al. review the state-of-the-art regarding the role of circulating sphingolipids in lipid in metabolism. They discuss the effect of dietary fatty acids on circulating plasma sphingolipids and sphingolipids in lipoproteins. They discuss the effect of dietary fatty acids on circulating plasma sphingolipids and sphingolipids in lipoproteins. Dietary fatty acids were shown to differentially affect the sphingolipidomic composition of plasma lipoproteins. Diets rich in saturated fatty acids were shown to increase long-chain ceramides in both plasma and lipoproteins in humans. These effects on circulating sphingolipids were not observed with diets rich in unsaturated fatty acids; however, data on effects of long-chain omega-3 fatty acids on circulating sphingolipids in humans are limited. They also review the effect of dietary sphingolipids, including those from milk polar lipids, on circulating sphingolipids in post-prandial and fasting plasma of humans. Recent evidence suggests that dietary supplementation with milk polar lipids may reduce atherogenic long-chain ceramide species in serum and chylomicrons. Overall, they demonstrate that various dietary lipids can modulate circulating sphingolipids, which they suggest should be investigated as part of nutritional strategies to prevent cardiovascular diseases.

In another review, St Germain et al. summarize PE homeostasis and conditions where there is impairment of either the CDP-ethanolamine or phosphatidylserine decarboxylation biosynthetic pathways. In particular, the authors focus on genetic disorders that affect these pathways, such as Liberfarb syndrome, and how these diseases may provide knowledge about PE metabolism to identify and develop novel therapeutics. Indeed, the authors provide a rationale for the development and testing of novel therapeutics for patients deficient in PE. They suggest that a realistic approach may be to develop treatment plans consisting of drugs known to affect PE metabolism in conjunction with PE supplements. However, they also suggest increasing lyso-PE acylation *in vivo* as an ambitious target worthy of investigation due to its potential to restore PE homeostasis in patients with dysfunction in either the phosphatidylserine decarboxylase or Kennedy pathways.

Finally, *Litopenaeus vannamei* is a species of Pacific white shrimp native to Central and South America and is one of the world's most important aquaculture crustaceans. However, because of the lack of suitable nutrition, the reproductive performance of these shrimp is lower than the same species of shrimp captured in the ocean. Therefore, Liang et al. investigated the effect of different phospholipids diets containing 4% phospholipids provided from either egg yolk, soybean, or krill oil for 28 days on the ovary development of the broodstock of *L. vannamei*. Their findings show that all three diets influenced ovary development, but krill oil was a superior diet and achieved significantly higher yolk particle distribution, a higher gonadosomatic index, and increased sterol hormone secretion (e.g., estrogen). Lipidomic analyses also demonstrated that the krill oil enriched the lipid composition of the ovaries, which is important for ovarian development. Changes in gene expression relating to fatty acid and glycerophospholipid metabolism supported these data. Overall, the authors concluded that the inclusion of dietary phospholipids, in particular krill oil, can favorably improve the development of the ovaries of *L. vannamei*. Identifying diets to improve aquaculture is critical for food sustainability. However, further research is required to identify more sustainable sources of dietary phospholipids to improve ovary development in *L. vannamei*.

In conclusion, this Research Topic adds a wealth of new information to the growing literature regarding the important role of phospholipids and sphingolipids in health, disease, and metabolism. As Editors, we would like to express our gratitude to all the contributing authors, reviewers, and editors at Frontiers in Nutrition for their support of this Research Topic.

## Author contributions

All authors listed have made a substantial, direct, and intellectual contribution to the work and approved it for publication.
